# Climate change mitigation: thermal comfort improvement in Mediterranean social dwellings through dynamic test cells modelling

**DOI:** 10.1007/s40095-022-00498-1

**Published:** 2022-05-09

**Authors:** Carmen María Calama-González, Ángel Luis León-Rodríguez, Rafael Suárez

**Affiliations:** Instituto Universitario de Arquitectura Y Ciencias de La Construcción, Escuela Técnica Superior de Arquitectura, Universidad de Sevilla, Av. Reina Mercedes 2, 41012 Seville, Spain

**Keywords:** Social housing stock, Thermal comfort, Climate change, Passive strategies, Mediterranean area

## Abstract

Global warming will lead to adverse consequences for human health and well-being. This research ought to determine whether passive low-cost strategies freely controlled by users (ventilation strategies, solar shadings or window operation) could be applied in low-income dwellings to meet acceptable thermal comfort to retrofit the Mediterranean social housing stock of southern Spain towards climate change. On-site measurements registered in some test cells (controlled environment with no users’ influence) were used to calibrate dynamic energy simulation models. The impact of several future periods, climate zones of southern Spain and orientations on thermal comfort was assessed. The results show that climate change triggers a more significant increase in outdoor temperatures in summer than in winter. Should ventilation be kept to minimum and blinds opened during daytime in winter, higher comfort would be achieved, with great differences between orientations and south reporting the best results. The higher the outdoor temperatures due to climate change, the higher the percentage of comfort hours (i.e. 23–68% in the present and 50–75% in 2080). In summer, natural night ventilation and blinds closed during daytime lead to the best comfort result, with negligible temperature differences between orientations. Future climate change scenarios worsen the percentage of comfort hours (i.e. 96–100% in the present, while up to 17% in 2080). Mechanical ventilation and blind aperture schedules were found to have the highest influence on overheating discomfort. Likewise, mechanical and natural ventilation schedules had the highest impact on undercooling discomfort.

## Introduction

Should the current anthropogenic activity rates be maintained, in all likelihood climate change will reach 1.5 °C in 2030–2052 and 4.8 °C in 2081–2100, becoming a severe threat to environmental systems and human population [1]. Climate change is generally believed to lead to more frequent and intense extreme cold and hot climate episodes, especially with strong urban heat effects [2], which may cause serious effects on human health and even increasing mortality rates [3]. To tackle this issue, low-carbon emission and energy targets are demanded by international agencies and governments, with an especial focus on the building sector, which is among the top-three dominant energy consumers in 2019 [4]. In fact, the existing European residential building stock accounted for almost 27% of the final energy consumed in 2018 [5]. Given that the new-built construction rate is slightly less than 2% and that three out of four buildings are energy inefficient [6], the improvement in existing buildings energy performance is a key objective to save energy and reduce greenhouse gas emissions, but also a significantly complex task for guaranteeing a proper indoor environmental quality, especially in low-income households [7], due to high fuel and energy poverty. Hence, global warming impedingly derives in adverse health and well-being consequences for users [8], especially for social householders.

In the Mediterranean climate, several studies evaluate the performance of the existing housing stock under extreme weather phenomena. Santamouris and Kolokotsa [9] assess several mitigation strategies, focusing on the performance of thermal insulation implemented in residential buildings during overheating periods. Zinzi and Agnoli [10] determine the influence of heatwaves on the energy performance of Italian dwellings, through on-site measurements and energy simulation assessment, analysing cool and green roofs. Ascione et al. [11] analyse different retrofit solutions applied to typical villas in the cities of Athens and Naples, considering thermal insulation, solar protections, types of windows, building systems and renewable energy sources, to minimise energy consumption. Panagiotidou et al. [12] assess how the addition of thermal insulation to the building’s envelope, window replacement and implementation of high-performance HVAC systems may impact on a residential building in Greece, in terms of greenhouse gas emissions reduction. Lassandro and Di Turi [13] assess the resilience of a multi-storey residential building in Bari (Italy) under future climatic conditions when thermal insulation is added to its facades. De Masi et al. [14] study a single-family dwelling of Benevento (Italy) towards climate change, assessing the impact of window replacement, thermal insulation, green roof or external solar shading on energy demand.

To confront climate change in low-energy dwellings, passive energy solutions are generally implemented instead of active strategies in order to retrofit social buildings [15], given the low incomes of social householders. Furthermore, shading protections and cooling measures, such as mechanical or natural ventilation [16], are key to environmentally mitigate indoor overheating [17]. In fact, window shading systems have proven to noticeably reduce heat deaths during warm periods [18] and the use of inactive design methods, such as air infiltration, becomes a cheapest solution to achieve comfort in low-energy buildings, reducing energy consumption, especially in warm periods [19]. In this line, Pérez-Andreu et al. [20] assess the impact of several strategies (such as infiltrations, shading devices or natural ventilation) on heating and cooling energy demand under future climate change scenarios, through the analysis of a single-family building located in Valencia. Santamouris et al. [21] also study the influence of natural night-time ventilation on the cooling energy demand of around 200 residential buildings in Greece, under high-temperature periods. Simões et al. [22] assess the influence of several shading devices systems and solar wall components on heating and cooling energy demand in 13 different Mediterranean regions.

Even though a large extension of studies conducted have been focused on assessing energy performance of buildings, there is a generalized lack of heating, ventilation and air-conditioning (HVAC) systems in social dwellings of southern Spain because of users’ low economical resources [23]. Since the vast majority of social householders cannot afford to pay the operational costs of HVAC systems, thermal comfort ought to be reached making use of environmental resources [24] and passive strategies that improve the building’s thermal performance. Hence, the improvement in indoor thermal comfort displaces the common challenge of reducing energy demand and consumption in the social housing stock, so more extensive research on this topic is still required.

This research aims at addressing the literature gap detected on thermal comfort improvement in contrast to the most commonly energy-related approach. The study focuses on the analysis of the thermal performance of existing social housing stock of southern Spain (Mediterranean area), under future climate change scenarios, since warm areas will be more sensible to global warming when compared to heating dominated climates. Specifically, given fuel and energy poverty in the social buildings, passive and low-cost strategies easily operate by low-income householders are analysed under present and future global warming scenarios (2030, 2050 and 2080). Different orientations, several ventilation strategies, solar protections and window operation techniques are assessed and compared through calibrated dynamic energy models. The final objective is to determine whether these low-cost passive strategies are sufficient to reach acceptable thermal comfort conditions in the social stock of the Mediterranean area, considering different future winter and summer climate conditions of southern Spain.

## Experimental and dynamic simulations

**Fig. 1:**
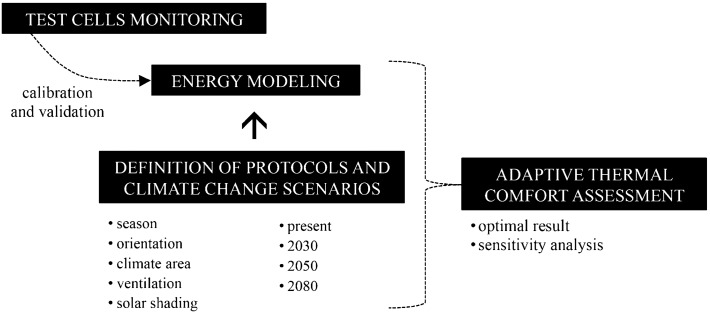
Scheme of the methodology followed

The methodology followed combines on-site monitoring and simulation modelling to assess the influence of different solar shading systems, ventilation protocols and window operational aspects on indoor thermal comfort in the social housing stock of southern Spain, considering current and future climate change scenarios. The steps are illustrated in Fig. 1.

### Step 1. Test cells monitoring and energy modelling

**Fig. 2:**
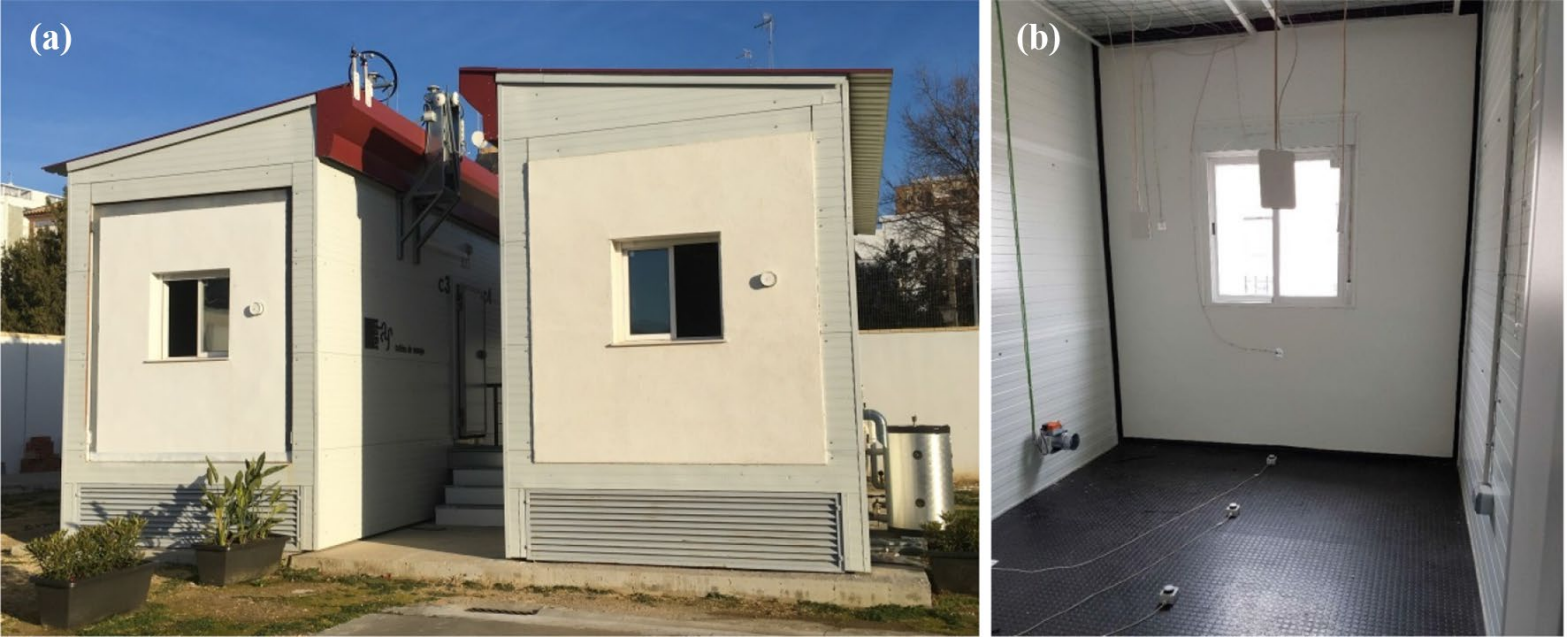
Test cells located in Seville (southern Spain): **a** general view; **b** indoors

On-site monitoring has been conducted in some test cells (Fig. 2), a highly controlled environment located in the city of Seville (southern Spain). This region is classified as Csa Mediterranean climate [25], with average maximum summer and minimum winter temperatures of around 30–35 °C and 5–6 °C, respectively. The test cells reproduce a housing space typical of the social dwellings of southern Spain and allow the controlled measurement of different ambient variables under real weather conditions and with no users’ influence. Later, an energy simulation model has been constructed with EnergyPlus 9.0.1 dynamic modelling open-access tool [26], incorporating occupation schedules during the year according to the Spanish regulations [27]. Later, using ambient hourly data recorded in the cells (indoor and outdoor temperatures, relative humidity and solar radiation values registered by a weather station placed on the roof of the cells) and following the procedure included in ASHRAE Guideline [28], the energy simulation model has been calibrated and validated. The calibration and validation process has been developed prior to the research presented in this paper and is extensively explained in [29]. Through this calibrated and validated case study, it has been possible to assess different climate areas of southern Spain and future climate scenarios.

### Step 2. Definition of climate change scenarios

Climate change scenarios have been selected according to the criteria established in the Special Report on Emissions Scenarios [30]. This report presents four future storylines, which depend on demographical, social, economic, technological and environmental developments. The first storyline, A1, corresponds to a combination of rapid economic growth, a global population increasing in mid-century and later declining and a rapid introduction of efficient technologies. The second scenario, A2, refers to a heterogeneous world with a continuously increasing global population, which fosters the preservation of local identities, a regionally economic growth and a slower introduction of technological changes. B1, which would be the third possible storyline, revolves around a convergent world, with a global population similar to A1. However, in this case rapid economic changes and efficient technologies will be introduced. Finally, B2 storyline establishes local and regional economic solutions, with a less rapid and more diverse technology and a global population similar to A2. According to the aforementioned report, these storylines are grouped into six future emission scenarios, based on the climate modelling approach, being all equally valid and with no assigned probabilities of occurrence: A2, B1, B2 and three scenarios of A1 with different energy technologies (A1F1: fossil fuel intensive, A1T: balanced, A1B: predominantly non-fossil fuel). A2 and B1 emission scenarios correspond to the most negative and positive approaches, respectively; meanwhile, A1B is an intermediate scenario [31]. Thus, in this research A2 scenario with three future periods (2030, 2050 and 2080) has been considered, which predicts up to 3–4 °C global average surface temperature change and average global carbon dioxide emissions slightly below 30 GtC/year.

With regard to the generation of 2030, 2050 and 2080 future climate conditions for the dynamic simulations, CCWorldWeatherGen 1.8 [32] has been used. This free-access tool takes into account the HadCM3 global climate model under A2 scenario, the worst emission scenario, from the Intergovernmental Panel on Climate Change (IPCC) [1], to convert present climatic conditions into climate change projections. Specifically, the EnergyPlus current weather file of the analysed region is imported to the CCWorldWeatherGen tool, which later exports the future climate projections as weather files (.epw). These files have been used in the energy simulation tool.

### Step 3. Definition of protocols and seasonal periods

In this stage, different ventilation techniques, solar shading protections and window operation protocols commonly implemented in the social housing stock of the Mediterranean area have been identified with the aim of determining their influence on the indoor thermal comfort of the social stock, under different climate conditions of southern Spain, future climate change scenarios and building orientations. The following subsections provide a more detail description of the aspects that have been considered in this research:

- Building orientation (axis). Simulations have been run under four orientations: south (S), north (N), east (E) and west (W).
- Ventilation. Two ventilation systems have been studied: mechanical and natural ventilation. For the first one, a mechanical extractor has been modelled, allowing the entrance of outdoor air into the cells with no additional thermal treatment. Two use schedules have been fixed: OFF and ON (continuous operation, according to the Spanish Technical Building Code regulations (Spanish Building Technical Code, 2019). For mechanical ventilation, three air ventilation rates have been considered: 0.5 ACH, 0.7 ACH (which is the minimum ventilation rate established by the Spanish Technical Building Code [33]) and 0.9 ACH (which would be a higher ventilation rate than the strictly required by building regulations, applied only in special cases, such as situations derived from COVID-19 pandemic). As regards natural ventilation, it occurs through the windows included in the building. In this case, through a 1.25-m^2^ sliding window with no thermal bridge, recreating the most typical ventilation conditions in the Mediterranean area or, in other words, a single-sided ventilation would be an unfavourable ventilation scenario. The natural ventilation rate was defined at 4 ACH in all cases, taking into account average wind values in the open space where the case study is located (recorded by a local weather station placed on the top of one of the test cells). In this case, four use schedules have been defined: OFF, ON during morning time (8:00 to 9:30), evening time (13:00 to 14:00) and night-time (22:00 to 8:00).
- Solar shading protections: External rolling PVC blinds have been considered, since it is a low-cost commonly used solar protection system in the Mediterranean area. Blinds operation have been defined under five possible use schedules: blinds totally opened (100%), blinds totally closed (0%), blinds half opened (50%), blinds opened at night (22:00 to 8:00) and closed during the day (8:00 to 22:00) and, finally, blinds closed at night (22:00 to 8:00) and opened during the day (8:00 to 22:00).
- Climate areas: The predominant climate areas of southern Spain have been analysed; in other words, the summer and winter climate conditions are more extended in Andalusia. The Spanish Technical Building Code Spanish Building Code [27] classifies southern Spain into several climate areas, depending on the solar radiation levels and degree-day. Specifically, winter and summer weather conditions are identified by two parameters: the climatic severity in winter (SCW) and the climatic severity in summer (CSS). The SCW is defined with a letter from “A” to “E”, “A” corresponding to milder winters and “E” to colder winters. On the contrary, the CSS is represented by a number from “1” to “4”, so that “1” refers to areas with milder summer and “4” to warmer summers. In this research, the most representative climate zones in southern Spain were considered, which are A, B and C in winter and 3 and 4 in summer. Furthermore, these areas also gather the highest number of social housing buildings [34].
- Seasonal periods: Since indoor thermal comfort assessment requires high-resolution data (i.e. hourly basis), two representative weeks have been analysed in all scenarios and protocols: for the winter season, 2–8 February, and from 15 to 21 July for summer.

Table 1 summarizes the building and operational parameters considered in the simulations. The last column (optimization) shows which variables have been included as optimization parameters.

**Table 1:** Building parameters and operational aspects considered in the simulations

Parameter	Description	Optimization
Building orientation	South (S), north (N), east (E), west (W)	Yes. The optimal combination of these parameters has been obtained through a single-objective optimization approach
Natural ventilation schedule	OFF, ON morning (8:00–9:30), ON evening (13:00–14:00), ON night (22:00–8:00)	
Natural ventilation rate	0.0 ACH, 4.0 ACH	
Mechanical ventilation	OFF, ON (continuous)*	
Mechanical ventilation rate	0.0 ACH, 0.5 ACH, 0.7 ACH*, 0.9 ACH	
Blinds schedule	Totally opened (100%), totally closed (0%), opened at night (22:00–8:00) and closed during day (8:00–22:00), closed at night (22:00–8:00) and opened during the day (8:00–22:00)	
Climate severity in winter*	A, B, C	No. These parameters refer to different scenarios that have been analysed
Climate severity in summer*	3, 4	
Climate projections	Present, 2030, 2050, 2080	
Occupation (0.024 people/m^2^)* schedule	For Weekdays SummerDesignDay, WinterDesignDay and AllOtherDays, Until: 07:00, 1/Until: 15:00, 0.25/Until: 23:00, 0.5/Until: 24:00, 1 For: Weekends and Holidays, Until: 24:00, 1	No. These parameters are input fixed data
Internal loads (2.2 W/m^2^)* schedule	For: AllDays, Until: 07:00, 0.1/Until: 18:00, 0.3/Until: 19:00, 0.5/Until: 23:00, 1/Until: 24:00, 0.5	

^*^Means “according to the Spanish Building Technical Code [27, 33]”

### Step 4. Present and future adaptive comfort assessment

Indoor thermal comfort assessment has been carried out considering the statistical adaptive model established in EN 16,798–1:2019 [35], which may be applied to buildings under free-running conditions and where users can freely control windows operation and modify their clothing level. This adaptive model can be implemented whenever average outdoor running temperatures are over 10 °C and below 30 °C. It is important to highlight that this model considers metabolic rates and thermal resistance of 1.0–1.3 met and 0.5 clo in summer and 1.0 clo in winter. The adaptive comfort temperature (T_com_) is determined from the running mean dry outdoor temperature for today (T_o,ref_), based on the daily mean dry outdoor temperature for previous 1 to 7 days (T_o,ref1_ to T_o,ref7_) (Eqs. 1 and 2). Three acceptability ranges may be defined based on the building category. In this study, a normal level of expectations has been considered (building category II, which equals a percentage dissatisfied < 10%), which corresponds to an adaptive comfort band of + 3 °C (upper limit) and –4 °C (lower limit).

1 $${\text{T}}_{{{\text{com}}}} = 0.{33} \times {\text{ T}}_{{{\text{o}},{\text{ref}}}} + {18}.{8}$$

2 $${\text{T}}_{{{\text{o}},{\text{ref}}}} = \left( {{\text{T}}_{{{\text{o}},{\text{ref1}}}} + 0.{\text{8 T}}_{{{\text{o}},{\text{ref2}}}} + 0.{\text{6 T}}_{{{\text{o}},{\text{ref3}}}} + 0.{\text{5 T}}_{{{\text{o}},{\text{ref4}}}} + 0.{\text{4 T}}_{{{\text{o}},{\text{ref5}}}} + 0.{\text{3 T}}_{{{\text{o}},{\text{ref6}}}} + 0.{\text{2 T}}_{{{\text{o}},{\text{ref7}}}} } \right){/3}.{8}$$

Considering the combination of the variables defined in Steps 2 and 3, the percentage of adaptive comfort hours (%) has been calculated for each scenario and protocol, based on the modelled indoor and outdoor temperatures reported by the simulation tool.

### Step 5. Optimization analysis

Conclusions have been obtained on how the most commonly used shading protections, ventilation and window operation protocols may influence thermal comfort in the social housing stock of the Mediterranean area, under the most representative climate areas of southern Spain, current and future climate projections (2030, 2050 and 2080) and building orientation (S, N, E, W). To do so, the parameter combination that reports the optimal thermal comfort conditions has been identified per each scenario and simulated case. Specifically, a single-objective optimization problem (only one objective function) has been considered; in other words, the percentage of thermal discomfort hours has been minimized. The aim is to find the global optima among all the possible combinations, which reports the minimum percentage of discomfort hours. The optimization problem has been computed using the non-dominated sorting generic algorithm (NSGA-II) [36] in the jEPlus + EA open-access software [37].

### Step 6. Sensitivity analysis on thermal comfort assessment

Finally, sensitivity analysis was performed with the aim of determining the influence on the overheating and undercooling discomfort caused by modifications in the variables considered. Specifically, the Morris method [38], later extended by Campolongo et al. [39], was chosen as screening technique given its wide implementation on building performance analysis and its adequate balance between low computation time and accuracy [40]. This one-step-at-a-time approach ranks the parameters in order of importance, considering their relative effect on the output results, through the consideration of the standard deviation (*σ*) (Eq. 3), which refers to the parameter’s interaction with other parameters, and the modified mean (μ*) (Eqs. 4 and 5), related to the parameter’s impact on the model output. In order to avoid cancellation effects derived from negative elements of non-monotonic models, the μ* proposed by Campolongo et al. [39] is considered, instead of the *µ*.

3 $$\sigma = \sqrt {\frac{1}{r}\cdot\mathop \sum \limits_{n = 1}^{r} \left( {{\text{EE}}_{n} - \mu } \right)^{2} }$$

4 $$\mu = \frac{1}{r}\cdot\mathop \sum \limits_{n = 1}^{r} {\text{EE}}_{n}$$

5 $$\mu^{*} = \frac{1}{r} \times \mu^{*} \mathop \sum \limits_{n = 1}^{r} \left| {{\text{EE}}_{n} } \right|$$

where *σ* is the standard deviation, *r* set of trajectories in which the space grid is sampled (independent EE), EE_*n*_ elementary effect (measures interactions with other parameters), *µ* mean of the value of the elementary effects and μ* modified mean of the finite distribution of absolute values of the EE.

## Results and discussion

**Fig. 3:**
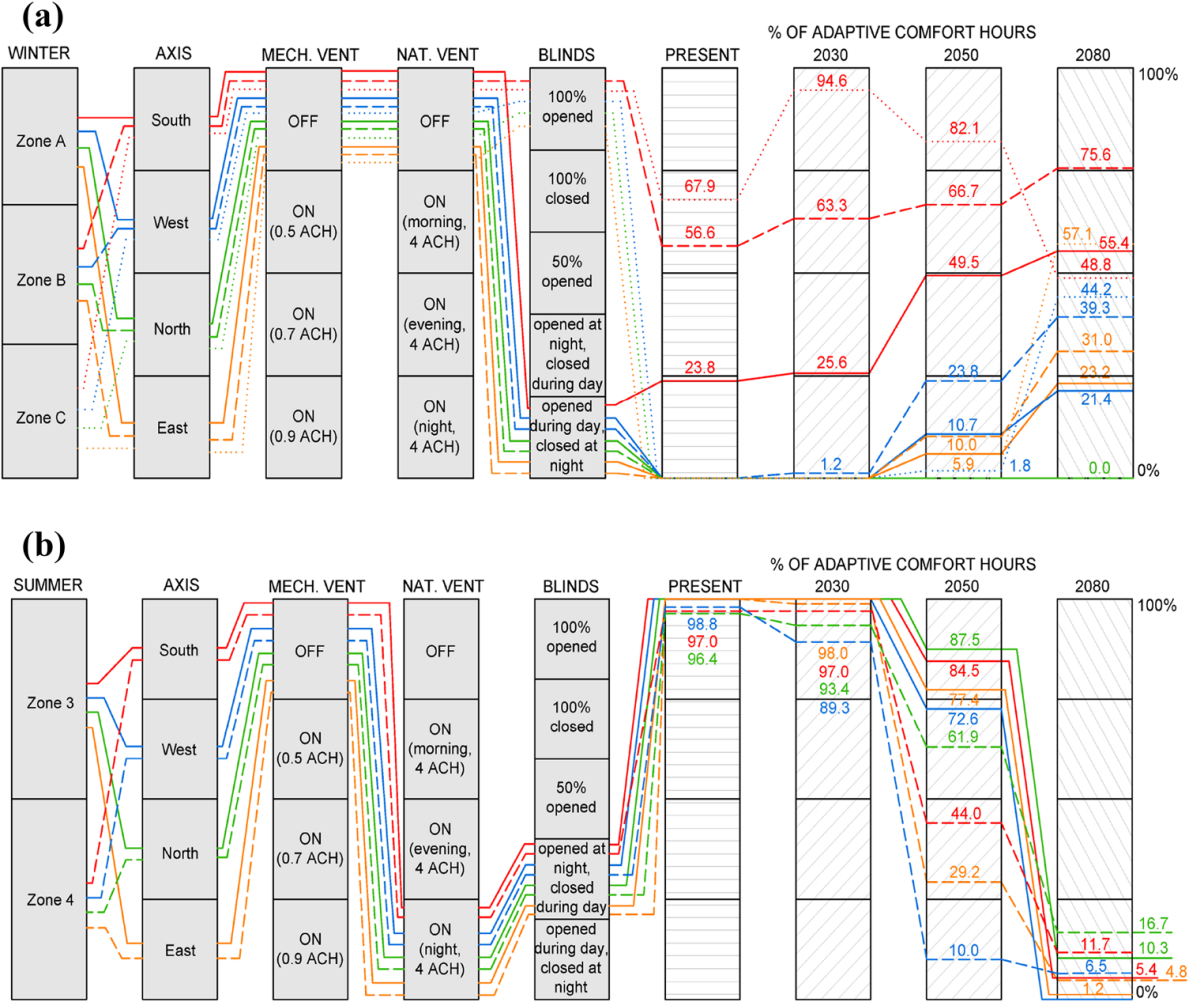
Parameter combination for the best thermal comfort result in: **a** winter; **b** summer

The parameter combination that reports optimal thermal comfort results for each protocol and scenario analysed is shown in Fig. 3, distinguishing between winter (A, B and C) and summer (3 and 4) climate areas of southern Spain. The specific percentage of comfort hours obtained for current and future climate projections (2030, 2050 and 2080) is also included.

In winter (Fig. 3a), generally the best parameter combination is ventilation OFF (both natural and mechanical) and blinds opened during the day and closed at night. There is an exception for A winter climate area in the south orientation: should blinds be kept totally opened, a higher percentage of comfort hours would be reported. The south orientation clearly reports the best comfort results, due to its higher solar radiation incidence when compared to the remaining orientations. These results significantly improved in 2080, given the increase in radiation levels. This fact occurs in A winter area, registering comfort hours up to 55.4%, and B winter zone, with approximately 75.5% comfort hours. Meanwhile, in C winter area, comfort decreases in 2050 in comparison with prior climate change scenarios, achieving comfort values below 50% of total hours in 2080. Notwithstanding, east and west orientations are noticeably improved in the C winter area under future projections, with better thermal comfort conditions than in A and B areas. In the north orientation, adaptive comfort standards are not meet in any scenario.

As regards summer period (Fig. 3b), the best combination of parameters is the same for all simulated cases, regardless of the orientation and climate area. Specifically, mechanical ventilation OFF, natural night-time ventilation (ventilation rate of 4 ACH) and blinds opened at night and closed during the day. Generally, comfort hours are almost over 90% in all cases for current and 2030 conditions. However, in 2050 and 2080, comfort percentages decreased, due to higher outdoor temperatures, which subsequently led to worse comfort results. This is dramatically worrying in 2080, with comfort values below 20%.

The following figures show the simulated indoor (T_in_) and outdoor (T_out_) temperatures for each climate area and weather scenario which correspond to the optimal comfort values previously reported, distinguishing between building orientations. The adaptive comfort band specific for the period analysed has been determined for each period and scenario and included into the figures.

**Fig. 4:**
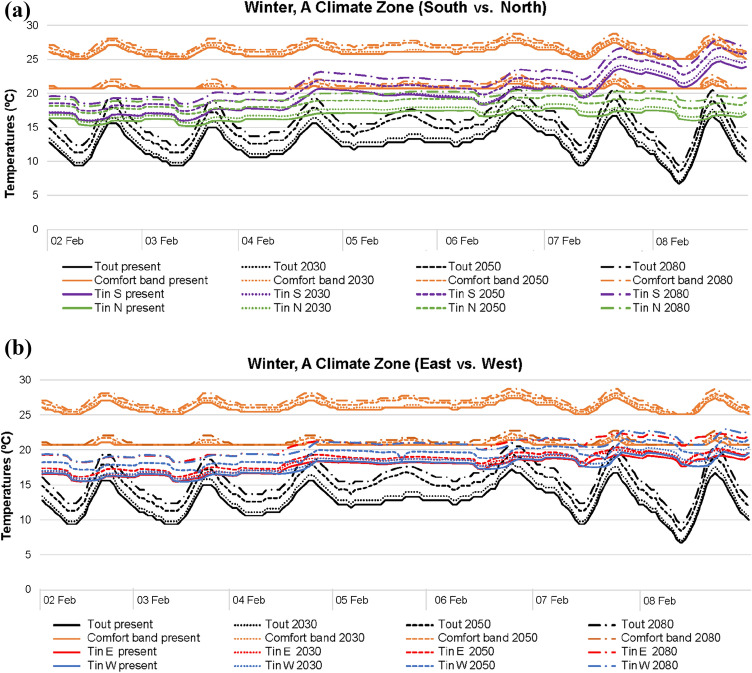
Temperatures in A climate zone for winter: **a** south and north; **b** east and west

**Fig. 5:**
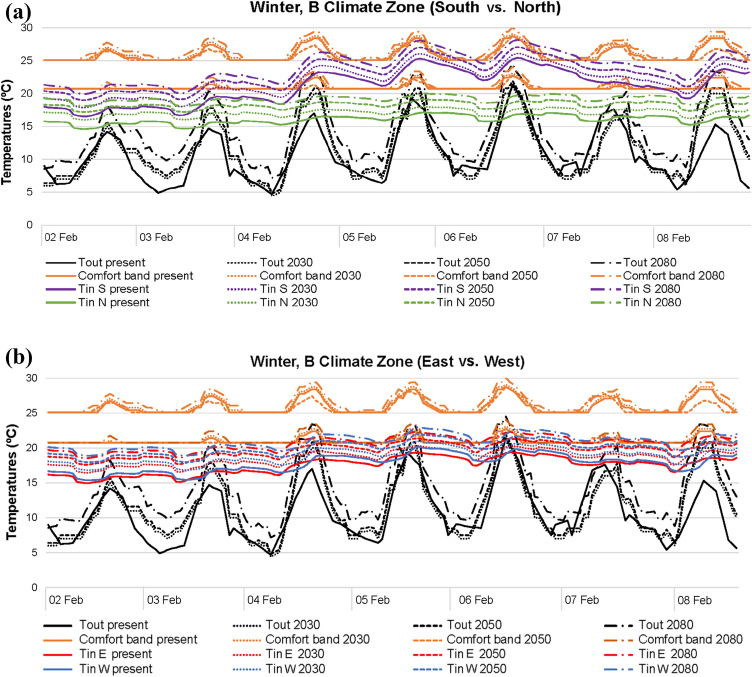
Temperatures in B climate zone for winter: **a** south and north; **b** east and west

**Fig. 6:**
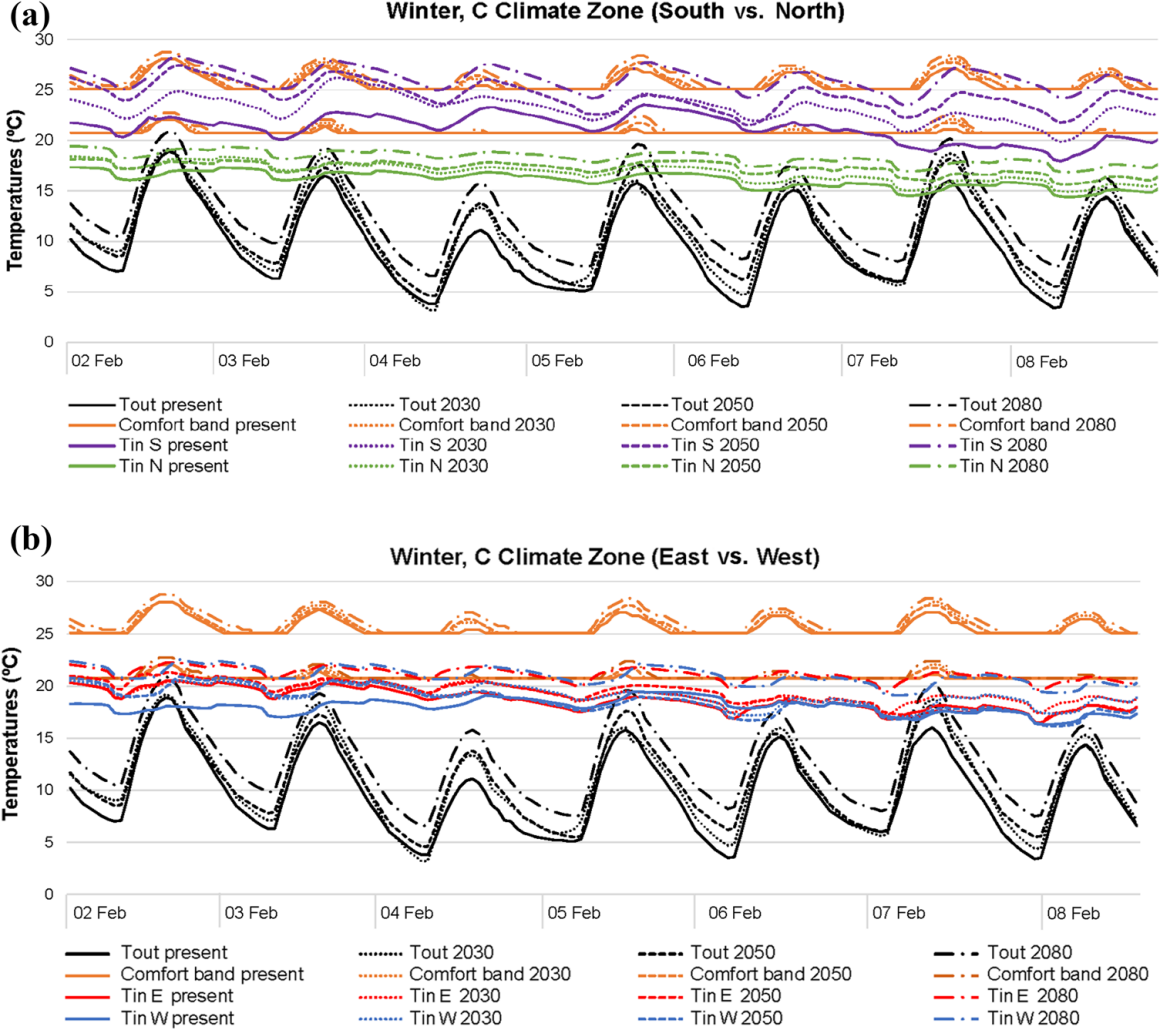
Temperatures in C climate zone for winter: **a** south and north; **b** east and west

In winter (Figs. 4, 5, 6), the higher the outdoor temperatures caused by future climate scenarios, the higher the indoor temperatures. This fact is quite significant in the south–north orientation, which reports temperature differences higher than those in the east–west. In general, the south orientation records the best thermal conditions. However, noticeably differences are also registered when A, B and C climatic areas are compared. A and B areas achieve comfort values with outdoor temperatures above 15–20 °C, while C area even punctually reaches comfort with outdoor temperatures below 10 °C. In comparison with the current climate scenario, 2080 clearly improves comfort conditions in all winter areas. Yet, north is the worst orientation in all climate scenarios and areas, since the lack of solar radiation results in lower indoor temperatures compared with the remaining orientations.

When outdoor temperatures are over 20 °C, temperature differences between south and north are higher, regardless of the winter areas. Meanwhile, should outdoor temperatures up to 15 °C be considered, both south and north orientations report similar indoor thermal conditions. Generally, in east, west and north of all current and future scenarios, indoor temperatures are normally within 15–20 °C, with outdoor thermal conditions ranging around 5–20 °C. With the same outdoor range, the south orientation tends to report indoor temperatures approximately of 20–25 °C, although there may be some punctually higher values. The reason for this is referred to the higher solar radiation influence of the south orientation, given that in this case, blinds were kept open during the day.

It is important to highlight that although optimal comfort results in winter were reported under no ventilation, this may derive in poor indoor air quality, with severe repercussions on users’ health, well-being and mood. Thus, to minimize risks caused by inadequate indoor air quality, especially given current pandemic circumstances due to COVID-19, the ventilation rate that led to the best results was implementing a 4-ACH morning-time natural ventilation (8:00 to 9:30) for south orientation and during evening time (13:00 to 14:00) for east and west orientations. Nonetheless, no improvement was reported for the north. These ventilation protocols achieved between 5 and 10% less percentages of adaptive comfort hours for all climate zones and future scenarios.

**Fig. 7:**
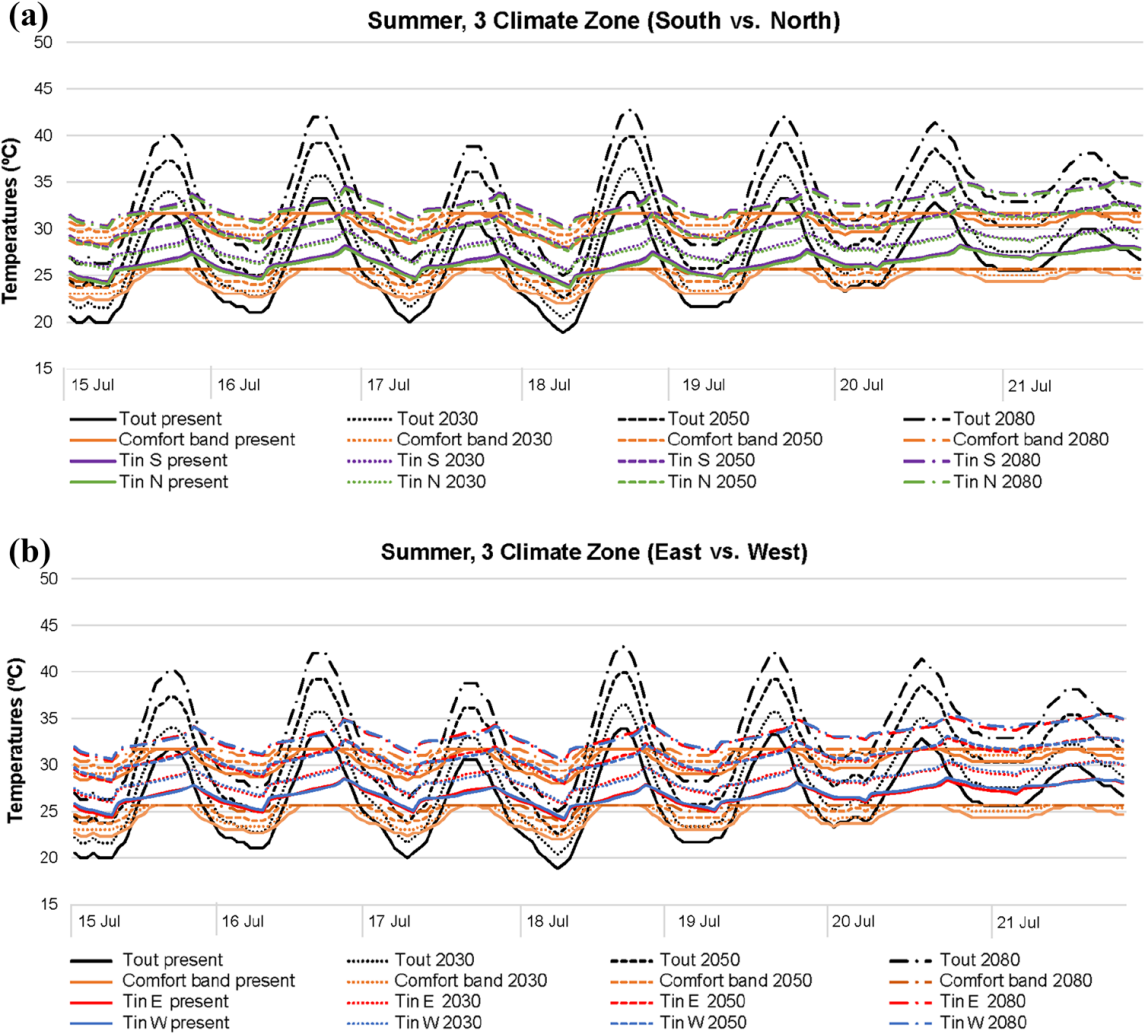
Temperatures in 3 climate zone for summer: **a** south and north; **b** east and west

**Fig. 8:**
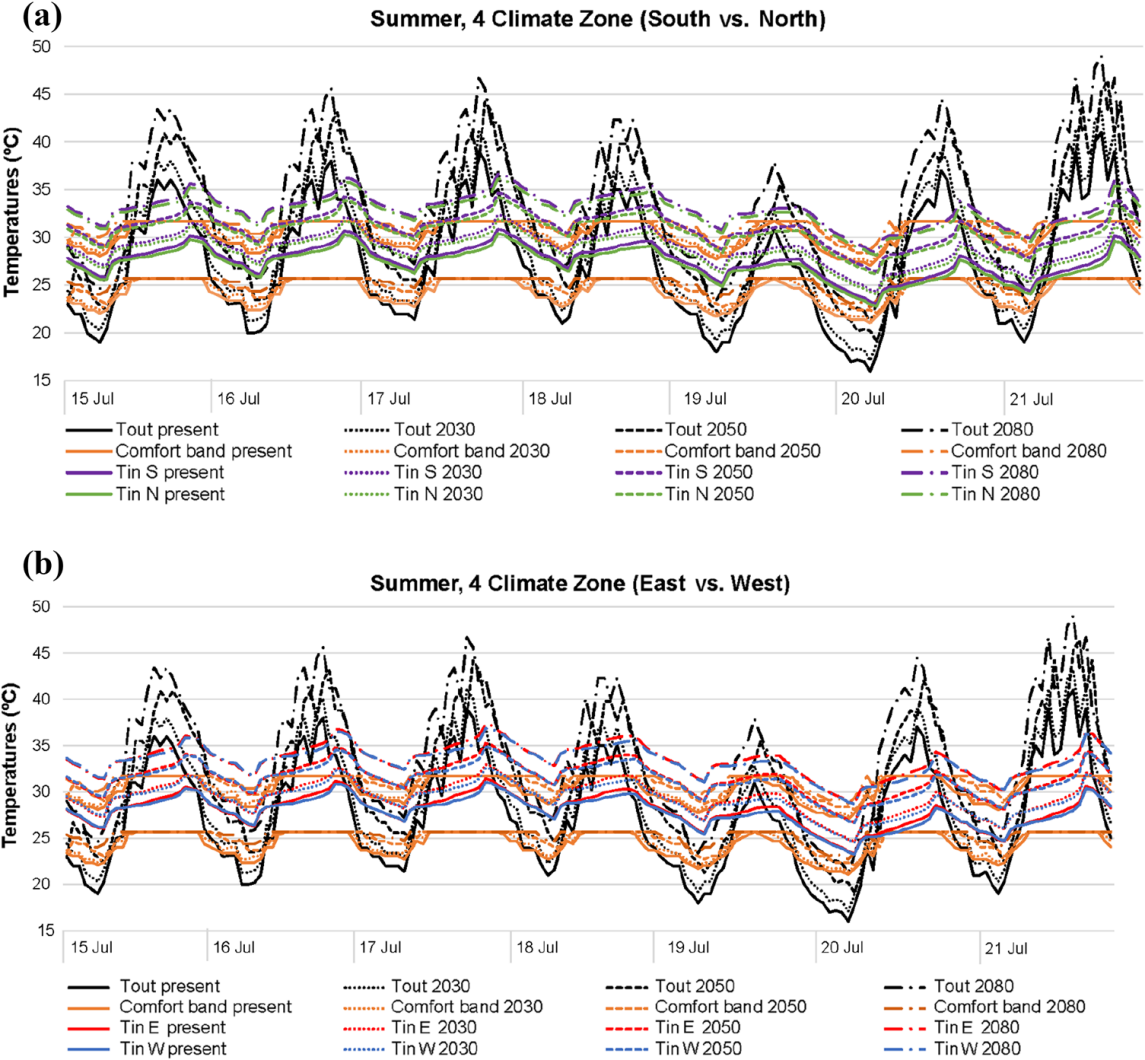
Temperatures in 4 climate zone for summer: **a** south and north; **b** east and west

In relation to the summer season (Figs. 7 and 8), indoor thermal differences between south–north and east–west orientations are hardly noticeable, regardless of the climate area (3 or 4). Nevertheless, indoor temperature differences due to future climate scenarios are worth highlighting, being generally higher than those obtained in winter.

The impact of the rise of outdoor temperatures caused by climate change is more significant in summer than in winter, especially in terms of maximum outdoor temperature peaks. Consequently, the influence on indoor temperatures is higher in summer, directly triggering worst thermal comfort results. Modifying the future climate scenario in winter led to indoor temperatures closer to thermal comfort; however, the opposite case is reported in summer: even though minimum indoor temperature peaks normally meet the comfort band, maximum indoor temperature peaks are not generally within comfort.

When considering all summer future climate projections and orientations, indoor temperatures are approximately 25–30 °C in both climate areas, punctually registering values up to 35 ºC, with outdoor temperatures of around 20–45 °C. The answer to this lies in the fact that in these cases blinds are closed during the day, decreasing direct solar radiation, and night-time natural ventilation is scheduled, reducing indoor overheating during the night.

**Fig. 9:**
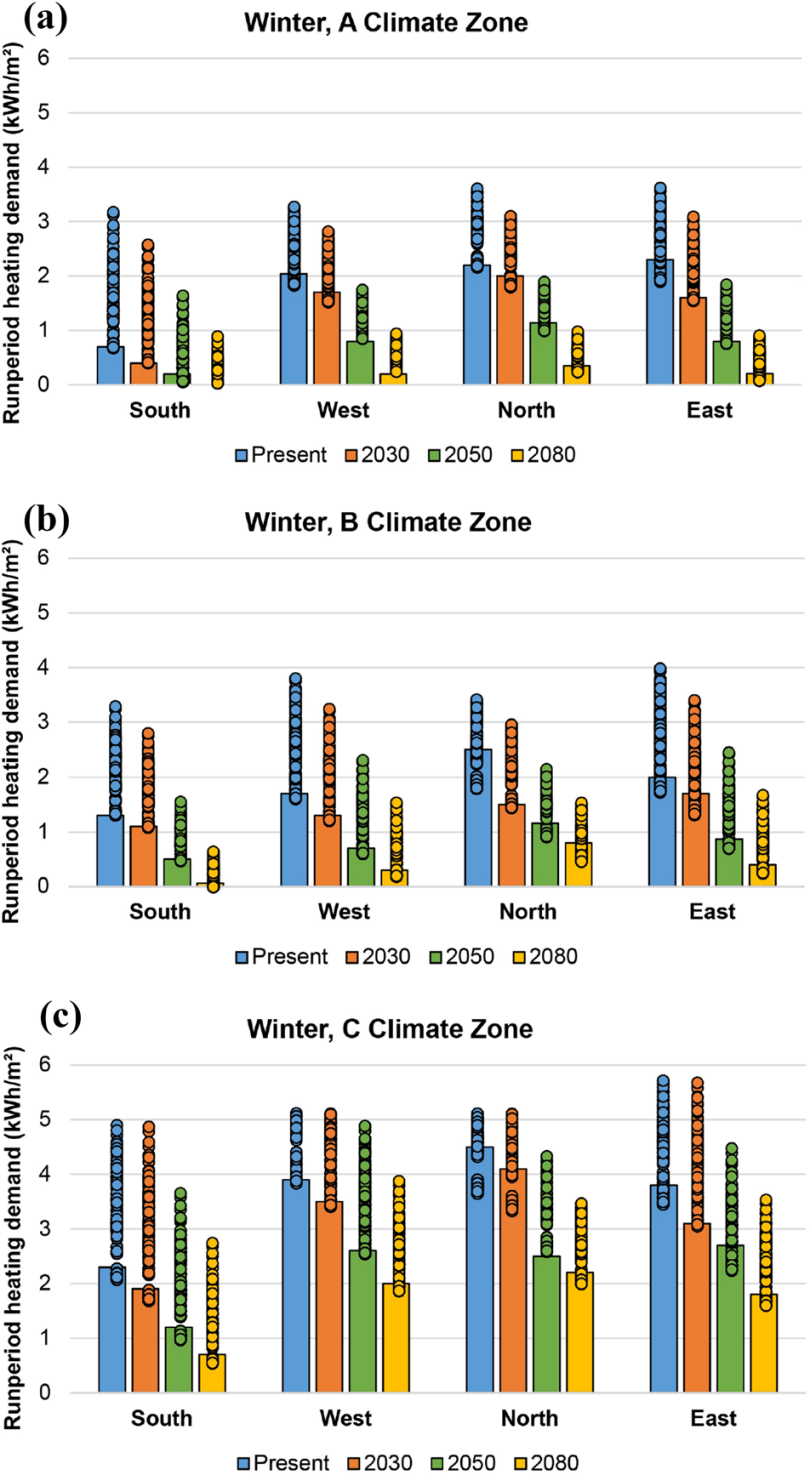
Heating demand (kWh/m^2^) during winter run period, per climate zone: **a** A, **b** B and **c** C

**Fig. 10:**
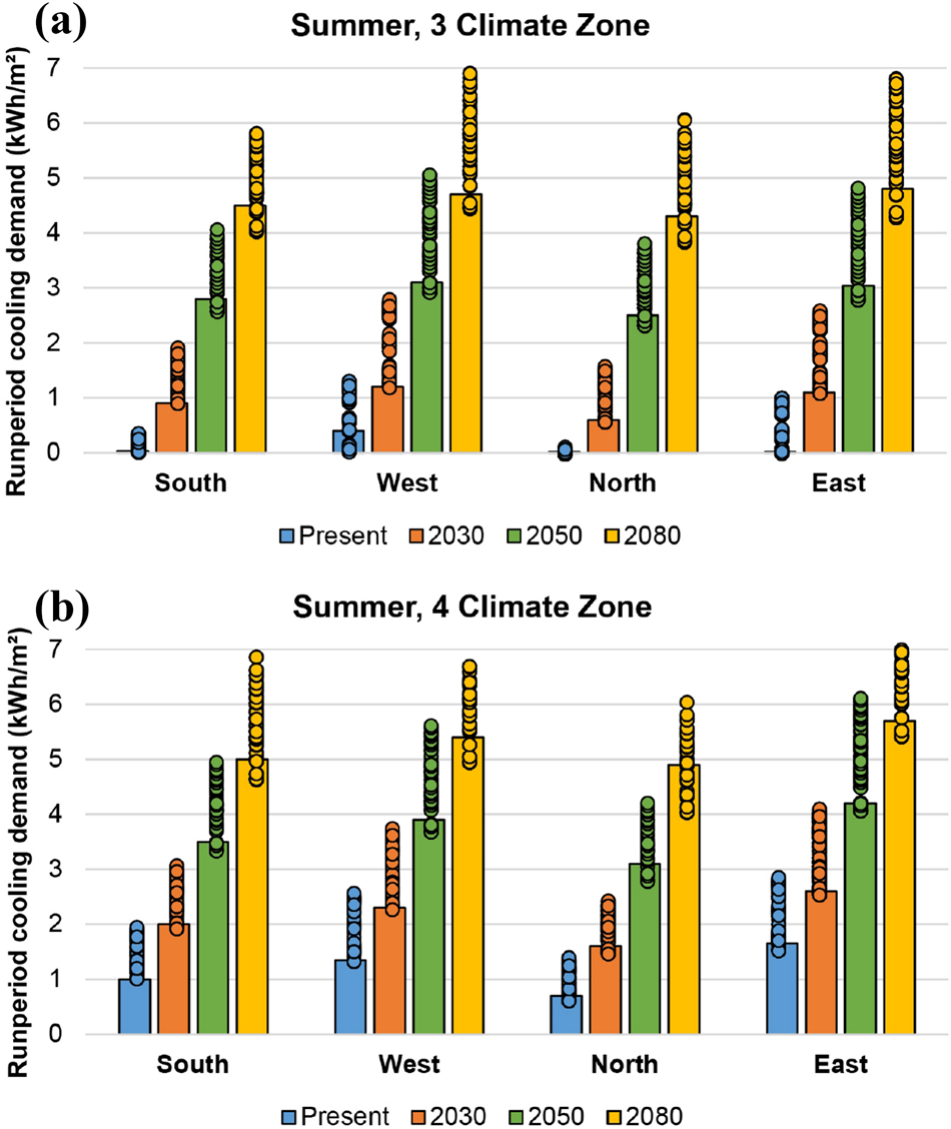
Cooling demand (kWh/m^2^) during summer run period, per climate zone: **a** 3 and **b** 4

Figures 9 and 10 show the results on energy heating and cooling demands (kWh/m^2^) reported in winter (A, B, and C) and summer (3 and 4) southern climatic areas, respectively. The demand values are presented per each building orientation and climatic scenario analysed (present, 2030, 2050 and 2080) and are referred to the specific run period used in each seasonal simulation (2 to 8 February in winter and 15 to 21 July in summer). The bars correspond to the best parameter combination, while the dots represent the values obtained in the remaining simulations.

It can be seen that heating demand (Fig. 9) decreases in all orientations proportionally to the future climate change scenario analysed, due to the increase in outdoor temperatures. The highest energy demand is recorded in the north, with values normally two times higher than those in the south. Meanwhile, east and west present an intermediate scenario in all winter areas, but with values noticeably close to those in the north. C climate area, given its greater climate severity in winter, registers the worst heating demands. Heating demand of the best parameter combination is among the best values obtained.

In summer (Fig. 10), the higher outdoor temperatures derived from climate change scenarios, the higher cooling demand in both climate areas (3 and 4). In this case, although the north orientation reports the best values given the lack of direct solar radiation, cooling demand is significantly similar in all orientations. It has to be highlighted that, considering the adaptive thermal comfort model, cooling demand in the present scenario is hardly noticeable in the south, north and east orientations of summer 3 climate zone. Once again, cooling demand of the best parameter combination is among the lowest values simulated.

**Fig. 11:**
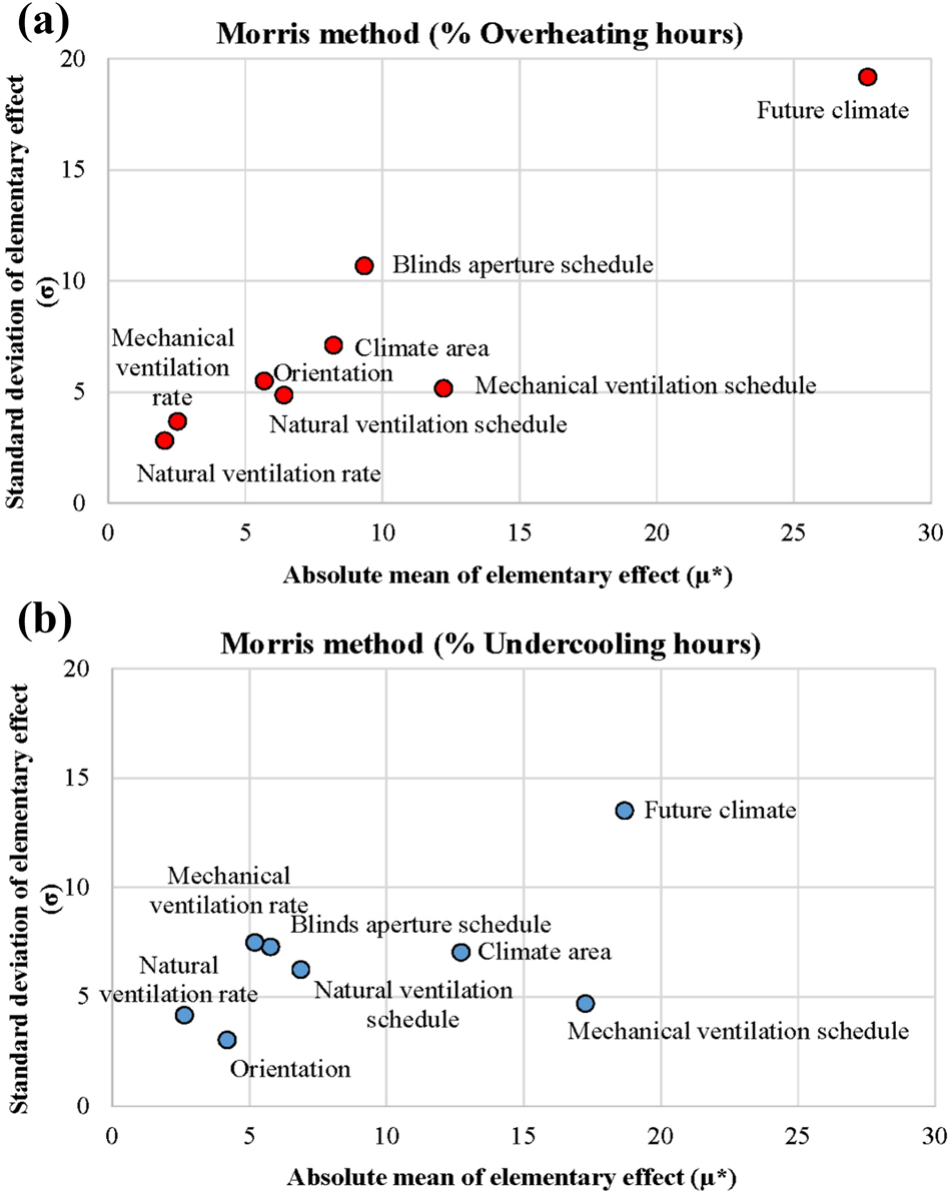
Sensitivity analysis on thermal discomfort: **a** overheating, **b** undercooling

Figure 11 presents the results of the sensitivity analysis conducted on the percentage of annual overheating and undercooling hours. Only variables explained in subsection 2.3 are considered: natural and mechanical ventilation rates, natural and mechanical ventilation schedules, orientation, blinds aperture schedule, climate area and future climate change conditions.

It can be seen that the most influential variable on both overheating and undercooling is the future climate (weather conditions). Thus, despite the retrofit strategies proposed, global warming will still have a significantly high impact on thermal comfort. Natural ventilation and mechanical ventilation rates are the variables with the lowest influence on the comfort results for overheating hours in the analysed case. Meanwhile, natural ventilation rate and orientation are at the bottom when undercooling discomfort is considered. The results also show that mechanical ventilation and blinds aperture schedules are key strategies to confront overheating discomfort in the social housing stock. Likewise, optimizing mechanical and natural ventilation schedules are of the utmost importance to tackle undercooling discomfort in the aforementioned stock.

Considering similar parameters as those analysed in the presented sensitivity analysis, Escandón et al. [41] use the standard rank regression coefficients method to conclude that natural ventilation rate and building orientation have a more significant impact on thermal discomfort than other variables, such as envelope parameters. Moreover, Gou et al. [42] implement Monte Carlo analysis to state that window opening factor, related to air infiltration due to natural ventilation, is of the utmost importance for indoor thermal comfort. Calleja et al. (2013) [43] determine that weather conditions were among the most influential parameters of building energy simulation, after analysing up to 130 variables. Similarly, Breesch and Janssens [44] highlight that the uncertainty on thermal comfort significantly increases when warm weather is applied in contrast to a standard weather data asset.

## Limitations and future work

Although ventilation rates considered meet the criteria required by Spanish regulations, future research should be conducted on the assessment of different ventilation rates, especially in terms of mechanical ventilation. This will allow comparing similar ventilation rates of both natural and mechanical systems. Likewise, including variable flow fans or mechanizing ventilation according to outdoor thermal temperature would be worth analysing.

Another highlighting fact is that EN 16798–1:2019 establishes a quite permissive adaptive comfort band for the Mediterranean area, especially in summer: for outdoor temperatures of 5–20 °C in winter and 20–45 °C in summer, comfort was generally reported with indoor temperatures of 20–25 °C in winter, but 25–30 °C in summer. Hence, more extensive research on different adaptive comfort models should be addressed to determine the more suitable ones for this area.

Furthermore, the comparison of the results under different future climate weather file generators, such as Meteonorm, or future emission scenarios may report interesting conclusions as well.

## Conclusions

Results show that the rise in outdoor temperatures caused by global warming is more significant in summer than in winter in the Mediterranean area. In winter, the best comfort results were reported with ventilation OFF and blinds opened only during daytime. The higher the outdoor temperatures, the higher the comfort hours, especially in the south. Yet, since this parameter combination may lead to inadequate indoor air quality, the optimal ventilated scenario, which included natural ventilation, reduces comfort hours by 10% in contrast to the non-ventilated case. In summer, the best results were reached with natural night-time ventilation and blinds opened only at night. Although comfort differences between orientations are less noticeable, they may be observed up to 2050. Yet, summer comfort hours are drastically reduced in 2080, regardless of the orientation. Winter and summer energy demand of the best parameter combination was relatively low compared with the other cases. Heating and cooling demand proportionally decrease and increase, respectively, under future climate change scenarios.

To confront overheating in the Mediterranean social housing stock, mechanical ventilation and blind aperture schedules were found to be key solutions. Likewise, mechanical and natural ventilation schedules have the highest impact on undercooling discomfort. Since these strategies are low-cost solutions (compared with the most commonly energy-related retrofit approaches, i.e. HVAC systems, envelope modifications, etc.), their optimization is key, given that they may be easily implemented in poor-energy dwellings and freely controlled by non-expert users, who may be more willing and enthusiastic to adequately incorporate them into their houses.
